# Diet–Microbiota Interplay: An Emerging Player in Macrophage Plasticity and Intestinal Health

**DOI:** 10.3390/ijms23073901

**Published:** 2022-03-31

**Authors:** Cian O’Mahony, Asma Amamou, Subrata Ghosh

**Affiliations:** APC Microbiome Ireland, College of Medicine and Health, University College Cork, T12 YT20 Cork, Ireland; aamamou@ucc.ie (A.A.); subrataghosh@ucc.ie (S.G.)

**Keywords:** intestinal inflammation, macrophage plasticity, gut microbiota, diet, short-chain fatty acid, indole, polyunsaturated fat

## Abstract

Inflammatory bowel diseases (IBD) are chronic disorders of the gastrointestinal tract with an increasing prevalence worldwide. Targeted therapies for IBD are limited by several factors, including the therapeutic ceiling and the high incidence of non-responders or loss-of-response. In order to improve therapeutic efficacy, there is critical need to decipher disease pathogenesis, currently not well understood. Macrophages, innate immune cells that exhibit high plasticity, perpetuate inflammatory signalling in IBD through excessive release of inflammatory mediators. In recent years, pioneering research has revealed the importance of the interplay between macrophages and gut microbiota in maintaining intestinal homeostasis. Particular attention is focusing on microbiota-derived metabolites, believed to possess immunomodulatory properties capable of manipulating macrophage plasticity. Microbiota-derived short-chain fatty acids (SCFAs) and indole compounds, along with dietary sourced omega-3 (ω-3) polyunsaturated fatty acids (PUFA), exert anti-inflammatory effects, attributable to interactions with macrophages. Before we can effectively incorporate these metabolites into IBD therapies, a deeper understanding of microbiota–macrophage interactions at a molecular level is necessary. Therefore, the aim of this review is firstly to detail current knowledge regarding how diet and microbiota-derived metabolites modify macrophage plasticity. Later, we discuss the concept of therapeutic strategies directed at microbiota–macrophage interactions, which could be highly valuable for IBD therapies in the future.

## 1. Introduction

Inflammatory bowel disease (IBD), including Crohn’s disease (CD) and ulcerative colitis (UC), are chronic inflammatory disorders with considerable morbidity posing major burdens on healthcare systems [1]. Despite extensive research, the aetiology of IBD still remains elusive, which complicates efforts for developing effective treatments. Macrophages are indispensable components of the innate immune system that form a front-line barrier against pathogens [2]. Under normal circumstances, macrophages phagocytose pathogens, dead cells, and cell debris, which primes the cell for orchestrating responses to tissue damage and infection [3,4]. However, impaired macrophage function perpetuates inflammation in IBD through excessive release of pro-inflammatory mediators, leading to the recruitment of T lymphocytes to the site of injury, eventuating as extensive tissue damage [5]. Under these circumstances, tissue repair mechanisms become overwhelmed, leading to the build-up of fibrotic tissue depositions, which further impairs organ function. These manifestations are partly driven by macrophage-mediated release of pro-fibrotic factors, leading to excessive fibroblast and myofibroblast activation. As intestinal fibrosis currently has no specific treatment, therapeutic approaches to restore normal macrophage activity may have a knock-on effect by halting progression of tissue fibrosis. Accordingly, macrophages are an attractive target for therapies aiming to alleviate intestinal inflammation and promote healing for individuals with IBD.

Macrophages can be categorised as having originated from blood monocytes, or otherwise, as being tissue-resident macrophages [6]. Macrophages in the first category—monocyte-derived macrophages—develop from monocytes circulating throughout the blood. These monocytes quickly migrate to the site of infection to undergo differentiation, where they provide replacements for depleted monocytes. In recent years, fate-mapping studies have characterised tissue-resident macrophages, which are derived from the foetal yolk sac during embryonic development and are embedded in their niche tissue before birth [7,8]. Uniquely, tissue-resident macrophages attain the somewhat limited capacity to replenish independently of blood monocytes [9]. Moreover, tissue-resident macrophages exhibit vast heterogeneity that varies according to ontogeny, tissue locality, and functional programming [10]. These characteristics permit macrophages to display differing phenotypes depending on the microenvironment that they inhabit.

Of particular interest to IBD research are intestinal-resident macrophages, which sense antigens derived from food and bacteria that breach the mucosal barrier of the alimentary canal [11]. Overall, intestinal macrophages constitute the largest pool of macrophages throughout the body and are described as being highly phagocytic, whilst remaining resistant to Toll-like receptor (TLR) stimulation [12,13].

Besides forming a first line of defence against pathogens, tissue-resident macrophages promote tissue healing and resolution following injury [12]. Therefore, in the mammalian gut, tissue-resident macrophages are imperative for maintaining intestinal homeostasis and function. In IBD, disturbances to epithelial barrier integrity lead to tissue-resident macrophages becoming overwhelmed by large infiltrations of the phenotypically different circulating monocytes, thus promoting a hyper-inflammatory intestinal microenvironment [13]. Under normal circumstances, it is believed that circulating monocytes soon transition toward a pro-healing phenotype to engage in tissue resolution activities. However, this phenotypic transformation is likely disrupted in IBD, leading to the continuance of inflammatory signalling and tissue destruction. Accordingly, targeting interplay between macrophages and the microbiota—an interaction believed to dampen gut inflammation—is a novel approach that possesses enormous potential for restoring intestinal function in IBD.

Over 1000 species of bacteria colonise the intestinal microenvironment, and many are pivotal in maintaining a homeostatic equilibrium. In the gut, microbiota reciprocate with the host for providing energy by helping to maintain intestinal barrier integrity and by defending against invading pathogens. Accordingly, dysbiosis of the intestinal microbiota has been implicated in a multitude of diseases [14]. Recent advancements in our understanding of these microbiota have propelled host–microbiota interactions to the forefront of medical research [15]. The most prevalent microbiota species include *Actinobacteria*, *Firmicutes*, and *Proteobacteria*, while populations of *Fusobacteria*, *Spirochetes*, and *Verrucomicrobia* are also present in lesser abundance [16].

**Figure 1:**
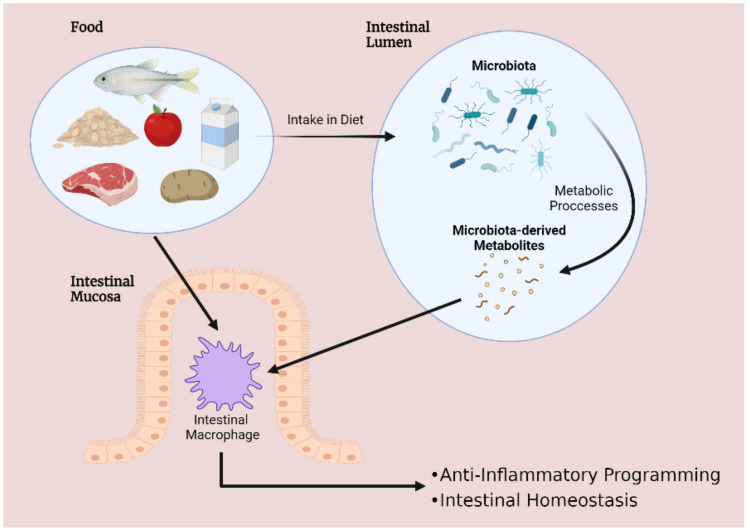
Interplay between diet, microbiota and intestinal macrophages encourages gastrointestinal homeostasis. Disruptions to this delicate balance are implicated in a wide range of disorders—including inflammatory bowel disease.

These microbial species interact intimately with the food products that are consumed, and microbiota composition varies according to dietary intake. Of particular significance is the breakdown of food by the microbiota to release an abundance of metabolites into the gut lumen. These processes give rise to two primary outcomes that are critical for intestinal homeostasis: altering gut microbial diversity and transforming the inflammatory signature of intestinal cells. Firstly, dietary patterns can reshape the microbiota by encouraging or inhibiting the growth of certain species. Alternatively, several foods and microbiota-derived metabolites exert immunomodulatory effects on intestinal cells, such as inducing changes to macrophage phenotype—an event commonly described as modifying macrophage plasticity (Figure 1). Considering the importance of microbiota–macrophage crosstalk, disruptions to this communication can have devastating consequences for the host. Notably, interactions leading to excessive activation of pro-inflammatory cytokine release can inflict damage to epithelial cells and induce necrosis, manifesting as IBD [15]. Therefore, several dietary approaches aiming to enhance microbiota–macrophage interactions are being considered as interventions to complement targeted therapies for IBD. Moreover, given that evidence points toward macrophages constituting major players in mediating intestinal immunity, studies concerning the interplay amongst microbiota and macrophages may provide novel insights into factors underpinning IBD pathogenesis. A deeper understanding of this topic could enlighten us toward the development of novel medicines for IBD.

## 2. The Role of Macrophages in the Development and Progression of Intestinal Inflammation

### 2.1. Macrophage Plasticity in Inflammatory Bowel Disease

Macrophages are multifaceted, highly dynamic cells that exhibit vast heterogeneity, reflecting both the tissue microenvironment that they occupy, along with the lineage that they are derived from [6]. In order to conduct specialised duties, macrophages exhibit a high degree of plasticity and display specific phenotypes, permitting the macrophage to adapt to the surrounding microenvironment. Intestinal-resident macrophages for instance, reside in the lamina propria, enabling the macrophage to sample extracellular surroundings and process antigens that they encounter. These surveillance duties facilitate a myriad of functions and defence mechanisms. Therefore, interactions of intestinal macrophages with microbes or foods could be viewed as critical events in shaping macrophage plasticity. For example, the tissue microenvironment could be expected to modulate cellular plasticity by virtue of a combination of a diverse cytokine milieu and environmental cues. This notion has been highlighted in murine studies, where pro-inflammatory macrophages that display high expression of Ly6C (LyC6^hi^) are reported to undergo in situ functional switches to pro-restorative LyC6^low^ macrophages in response to the surrounding environment [17].

Broadly speaking, in vitro macrophage plasticity is commonly classified according to the broad categorisation of being inflammatory M1 macrophages or the anti-inflammatory M2 macrophages. While transitions between macrophage phenotypes have been well-defined in vitro, labelling of macrophage populations is convoluted in vivo due to the complexity of macrophage heterogenicity. Specifically, evidence suggests that macrophage plasticity exists as part of a spectrum, where M1 and M2 represent polar ends [12]. M1 macrophages exhibit several distinguishing features that help underpin a pro-inflammatory phenotype. First of all, pro-inflammatory M1 macrophages are induced through stimulation with lipopolysaccharide (LPS) and interferon gamma (IFN-γ), leading to secretion of tumour necrosis factor alpha (TNF-α), interleukin 1 beta (IL-1β), interleukin 6 (IL-6), and interleukin (IL-12) to encourage microbicidal activity and phagocytosis [3]. These robust immune responses are energy demanding, and in recent years, it has become evident that macrophages undergo metabolic reprogramming in response to infection, which dictates the ability of the macrophage to conduct rapid responses to microbial invasion [18].

**Figure 2:**
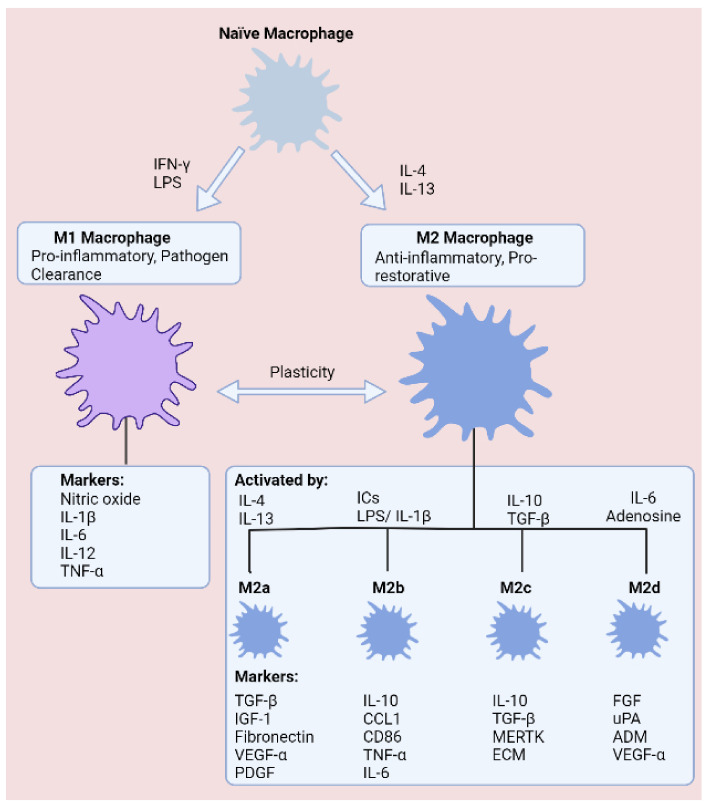
States of macrophage polarisation. Macrophages are commonly defined as classically activated (LPS and IFN-γ) M1 macrophages or alternatively activated (IL-4 and IL-13) M2 macrophages, which comprise four subgroups (M2a–M2d). Macrophages exhibit high plasticity, enabling transitions between phenotypes according to the surrounding tissue microenvironment.

Given the necessity for almost-instantaneous responses to prevent microbial dissemination within the body, M1 macrophages are understood to upregulate glycolysis during polarisation as a means of supporting vigorous immune responses and structural changes. In respect of recruiting immune cell populations and enhancing microbial killing, M1 macrophages accumulate succinate during pro-inflammatory conditions, which stabilises HIF-1α and promotes secretion of IL-1β [19]. At the other end of the spectrum, M2 macrophages are instrumental in suppressing immune responses, minimising tissue damage, and resolving tissue healing following injury. In an alternative manner to M1 macrophages, the anti-inflammatory programming of M2 macrophages is fuelled through oxidative phosphorylation (OXPHOS) and fatty acid oxidation (FAO). OXPHOS is preferred on account of the abundance of ATP molecules produced relative to glycolysis, providing additional energy for tissue resolution. Understandably, FAO is another crucial metabolic pathway, as FAO generates acetyl-coA, thus helping to ensure maximum ATP generation from the tricarboxylic acid (TCA) cycle and OXPHOS. M2 macrophages have been further branched into the M2a, M2b, M2c, and M2d subtypes, which are classified according to cytokine secretion, expression of cell surface markers, and transcriptome and biological activities (Figure 2).

While M1/M2 nomenclature is a simplification, the fact of the matter is that equilibrium between these opposing phenotypes is critical for intestinal tissue homeostasis. Therefore, it is of high value that inflammatory signalling is subject to stringent regulatory mechanisms. Disruptions to this status quo may tip the balance toward autoimmunity, or conversely, may be deleterious by promoting immunodeficiency and an inability to clear harmful microbes.

### 2.2. The cGAS-STING Signalling Pathway: An Emerging Regulator of Macrophage Plasticity

A driving force behind perpetuated inflammation and tissue injury in IBD involves an intricate link between the innate and adaptive immune systems—namely, recruitment of T lymphocytes to the site of injury through the release of chemokines by macrophages. Therefore, pathways involved in mediating these responses are of high importance to unravel. Activation of the cyclic GMP-AMP synthase (cGAS)—stimulator of interferon genes (STING) signalling pathway, which promotes macrophage cross-priming—is reported to be crucial for bridging this link [20].

cGAS is a cytosolic dsDNA sensor that detects intracellular DNA derived from pathogens or damaged host cells. Upon recognising dsDNA, cGAS generates the secondary messenger 2′3′-cGAMP from ATP and GTP, which in turn activates the endoplasmic reticulum-localised STING protein. These interactions initiate a downstream signalling cascade culminating in activation of transcription factors nuclear factor kappa B (NF-ĸB) and interferon regulatory factor 3 (IRF3), stimulating the release of interferon-beta (IFN-β) and pro-inflammatory cytokines. Activation of the cGAS-STING axis in macrophages is an important initial step in the recruitment and activation of T lymphocytes, setting in motion clonal expansion of these cell populations. Later, these T lymphocytes become embodied as indispensable arms of the adaptive immune system. Indeed, heightened activation of macrophage cGAS-STING signalling has previously been associated with a high-fat diet, demonstrating an intricate link between diet and systemic inflammation. Moreover, a functional role of the cGAS-STING signalling axis extends to intestinal epithelial cells, where the pathway mediates protective effects by promoting intestinal barrier integrity [21,22].

**Figure 3:**
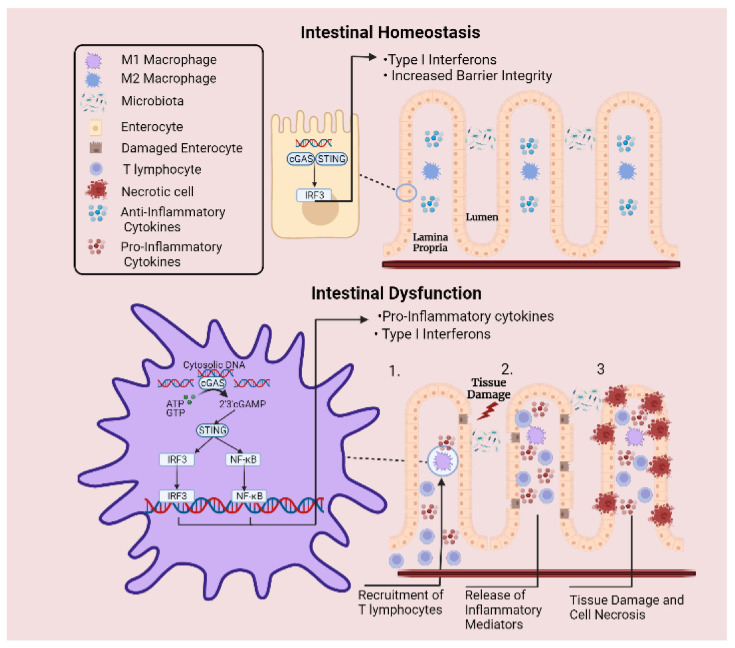
Involvement of cGAS-STING signalling pathway in intestinal inflammation. Under normal circumstances, cytosolic DNA is sensed by cGAS-STING signalling axis, which is involved in promoting intestinal barrier integrity. Meanwhile, excessive inflammation is prevented by cytosolic DNAse enzymes that degrade free DNA. However, under inflammatory conditions, the regulatory capacity of these enzymes is overcome when a DNA threshold is reached, leading to abnormal levels of cytosolic DNA. Upregulated expression of STING protein and elevated pathway activation culminates in sustained release of pro-inflammatory cytokines and type I interferons. (**1**) This mechanism activates the adaptive arm of the immune system and leads to the recruitment of T lymphocytes to the site of injury. (**2**) Perpetual release of inflammatory mediators by T lymphocytes leads to aggregations of immune cells and extensive release of inflammatory mediators. (**3**) Surrounding epithelial cells are damaged by the sustained inflammatory response, resulting in cell necrosis and tissue destruction.

Interestingly, recent studies have highlighted a potentially novel role for cGAS-STING in modulating macrophage plasticity (Figure 3). Specifically, in a DSS-induced model of murine colitis, elevated expression of STING protein was documented, leading to heightened sensitivity to the STING agonist: cyclic dinucleotide (CDN) [23]. As expected, the authors demonstrated that STING expression did indeed worsen colitis, whilst directing naïve and M2 macrophages in the direction of an M1 phenotype. Moreover, reactive oxygen species (ROS)-induced oxidation of DNA confers resistance to degradation and enhances activation of cGAS [24]. Despite previous studies denoting that cGAS-STING signalling may be detrimental in inflammatory disease, little is known regarding the extent to which the pathway controls macrophage plasticity in IBD. Indeed, it is plausible that dsDNA derived from host cells or microbiota may potentiate activation of cGAS-STING signalling in IBD. Moreover, whether excessive pathway activation promotes abnormal T lymphocyte migration to the intestinal microenvironment has not been studied. Nonetheless, pioneering discoveries in recent years emphasise the multi-functional basis of this DNA sensor. Importantly, these findings reveal another potential means of modifying macrophage plasticity in the gastrointestinal tract and encouraging homeostasis in the intestinal microenvironment.

## 3. Dietary Approaches for Targeting Macrophage Plasticity

While several studies have detailed associations between microbiota imbalances and IBD [25], others have defined the effects that microbiota-derived metabolites exert on immune cells. The microbiota generates an abundance of metabolites, and many of these enter systemic circulation, where they mediate modulatory effects upon reaching target tissues [26]. A better understanding of how these metabolites influence gut homeostasis could enlighten us toward ‘immunonutrition’ approaches, which encompass dietary interventions for circumventing excessive inflammation in IBD. Various foods are in the spotlight as candidates for immunonutrition by virtue of favourable interactions with the microbiota. Because of this important research, several metabolites have been highlighted that modify immune cell function, leading to suppression of intestinal inflammation and enhanced tissue healing. Notably, foods that give rise to high levels of short chain fatty acids (SCFAs), indole-compounds, and omega-3 fatty acids (ω3FAs) have been underlined as possessing immunomodulatory capacity. Whether these effects can be harnessed to manipulate macrophage plasticity, and thus control the inflammatory state of the cells, is a highly important question in medical research. Essentially, the microbiota could be viewed as a critical connection between food intake and downstream modulation of immune cell function. Accordingly, targeting the microbiota to regulate metabolite production could serve as a novel platform for the development of adjuvants for IBD therapies. In the following section we have highlighted three dietary-derived metabolites: SCFAs, indole-compounds, and ω3FA, which display distinct therapeutic promise as adjuvant therapies for treating IBD. 

### 3.1. Short-Chain Fatty Acids

Co-evolution has given rise to the emergence of symbiotic relationships between the host and commensals. The microbiota in the gut are chief examples that acquire energy from the food that passes along the intestinal track of the host. Microbiota reciprocate to this gesture by promoting intestinal barrier homeostasis, dampening inflammation, strengthening the epithelial barrier, and preventing colonisation by pathogens [27]. A predominant mechanism of conducting protective duties is via production of SCFAs, which are fatty acids containing fewer than six carbon atoms, the most common in the mammalian gut being acetate (C2), propionate (C3), and butyrate (C4) [28]. Intriguingly, SCFAs ameliorate disease activity in IBD and promote healing of the colon [29,30].

In the mammalian gut, microbiota generate SCFAs through fermentation of indigestible fibres. Accordingly, several high-fibre foods have been reported to induce favourable outcomes for reducing risk of and disease state of IBD. Remarkably, fibre exhibits several clinical benefits in IBD, such as prolonging remission and reducing lesions in the intestinal mucosa [31]. Imbalances in consumption of fruit and vegetables, both abundant in fibre, were reported to be a risk factor for the emergence of IBD [32,33]. Oats are another rich source of dietary fibre and have been shown to prevent deterioration of gastrointestinal symptoms in UC [34]. Likewise, high-fibre legumes, such as navy and black beans, are capable of attenuating intestinal inflammation in murine models of IBD [35]. Taken together, it is evident that intake of high-fibre foods is advantageous for maintaining intestinal health. Indeed, interplay between fibre and microbiota, leading to generation of SCFAs, is a critical event in this process.

SCFA concentration varies along the gastrointestinal tract, with mean concentrations reaching 13 mmol/kg in the terminal ileum, 80 mmol/kg in the terminal colon, and 131 mmol/kg in the caecum [36]. This disparity in SCFA concentrations is likely dependent on the diversity of microbial populations along different portions of the tract. Butyrate is primarily produced by the microbial phylum *Firmicutes* and shows highest concentration in the colon and caecum [37]. In particular, the bacteria *Faecalibacterium prausnitzii* and *Eubacterium rectale*, from the families *Ruminococcaceae* and *Lachnospiraceae*, respectively, are exceptionally robust producers of butyrate [38]. Alternatively, propionate and acetate are predominantly generated by *Bacteroidetes* in the small and large intestines [37]. Considering that production of SCFAs is predominantly conducted by certain bacterial populations, it no surprise that altered microbiota composition is commonly seen in individuals with IBD [39]. SCFA-producing *Butyricicoccus*, for example, are depleted in patients with active IBD [40].

Over the past decades, it has been well established that foods that generate high levels of SCFAs are protective against IBD. Indeed, several important pieces of evidence now support the hypothesis that these effects are at least partly resulting from the effects of SCFA on immune cells. Of particular significance are studies proposing that favourable outcomes are largely stemming from modulation of intestinal macrophages. A deeper understanding of these interactions, especially regarding the mechanisms that SCFAs can reprogram macrophages toward anti-inflammatory behaviour, would be an invaluable tool for IBD therapy.

**Figure 4:**
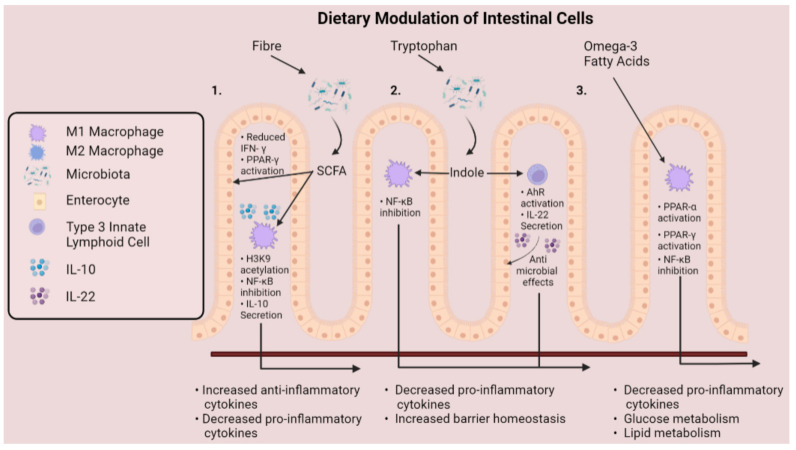
Mechanisms that dietary metabolites modulate cells of the gastrointestinal tract. Fibre and tryptophan are metabolised by the microbiota to release short-chain fatty acids and indole compounds into the gut lumen. (**1**). Short-chain fatty acids enhance PPAR-γ activation and reduce IFN-γ production of intestinal epithelial cells, while inducing immunomodulatory effects on intestinal macrophages. (**2**). Indole suppresses activation of NF-kB and stimulates production of IL-22 by group 3 innate lymphoid cells, promoting epithelial barrier homeostasis. (**3**) Omega-3 polyunsaturated fats promote an anti-inflammatory macrophage phenotype through activation of PPAR-α and PPAR-γ, along with inhibition of NF-κB, culminating as decreased production of pro-inflammatory cytokines.

Following production by the microbiota, SCFAs are available to interact with macrophages stationed along the intestinal mucosa. Luminal SCFAs are taken up by the cell through various means, including passive diffusion and carrier-mediated transportation or otherwise through binding to G-protein coupled receptors (GPR41, GPR43, and GPR109a) [41]. Following uptake by the cell, SCFAs mediate immunomodulatory changes to immune cells through several mechanisms [42,43,44,45]. The most studied SCFA—butyrate—exerts anti-inflammatory effects in IBD through inhibition of NF-ĸB activation, suppressing release of pro-inflammatory cytokines (Figure 4) [46]. In accordance, acetate and propionate exhibit comparable inhibitory properties with butyrate at suppressing NF-κB activation [47]. Indeed, while SCFAs also activate GPCR signalling, immunomodulatory effects are mediated independently of these receptors [42].

Anti-inflammatory effects mediated by butyrate have encouraged various laboratories to delineate molecular interactions underpinning these effects. Traditionally, inhibition of histone deacetylases (HDAC) has been believed to underlie the anti-inflammatory signature of butyrate [48]. Investigations by Chang et al., for example, determined that butyrate acts as an inhibitor of HDAC, which is an enzyme that regulates gene transcription by removing acetyl groups from histones. In the study, butyrate downregulated NO, IL-6, and IL-12 in intestinal macrophages, likely through enhanced histone acetylation [42]. These findings build on prior data showing that butyrate attenuates LPS-induced secretion of TNF-α, IL-1β, IL-6, and NO, whilst prompting the release of anti-inflammatory IL-10 in RAW 264.7 monocytes [45]. On the basis that butyrate is an HDAC inhibitor, studies have speculated that the SCFA induces epigenetic reprogramming [43]. In line with this concept, bone marrow-derived macrophage (BMDM) polarisation toward an M2 phenotype was induced by butyrate through acetylation of H3K9, which showed to be a histone modification that enhances STAT6 activation [49]. The question remains whether butyrate can modulate macrophage plasticity. Production of ROS, which are key regulators of an M1 phenotype, can be attenuated in neutrophils following butyrate treatment [50,51]. Therefore, it is plausible that butyrate exerts similar effects on macrophages. Collectively, these observations represent a molecular basis to explain why a high-fibre diet has a protective effect against intestinal disorders [31].

Ultimately, although SCFAs appear to be central in mitigating against intestinal injury, we have still not mapped the entire signalling landscape underpinning these effects. While evidence points toward butyrate activating G-protein coupled receptors (GPR41, GPR43, and GP109a) and regulating several immune and inflammatory processes [41], whether these receptors are involved in ameliorating inflammation in IBD has yet to be elucidated. A greater knowledge of these interactions could inform us of more appropriate targets for the treatment of IBD in the future. Moreover, while transcriptions factors are central in modulating macrophage plasticity [52], the true nature of interplay amongst SCFAs and transcription factors, and the ensuing impact on macrophage function, have not yet been clarified.

### 3.2. Tryptophan-Derived Metabolites

Tryptophan is an essential amino acid (humans cannot produce tryptophan endogenously), meaning instead that tryptophan must be supplemented in the diet. Many foods are good sources of tryptophan, including oats, milk, cheese, tuna fish, chicken, and turkey [53]. Amongst these tryptophan-rich foods, oats, lean meats, milk, and cheese are all associated with ameliorated intestinal inflammation in IBD [54]. 

Indeed, consumption of tryptophan in the diet is associated with intestinal homeostasis. Serum tryptophan levels, for example, are lower in individuals with IBD, denoting that tryptophan deficiency or degradation may exacerbate gut dysbiosis [55]. This notion extends to animal models of colitis, such as in mice, where mice deficient in tryptophan were recorded to display exacerbated colitis [56] (p. 2). From a medical standpoint, it is apparent that dietary tryptophan supplementation could represent a means of attenuating intestinal inflammation. This is supported by animal models, where dietary supplementation of tryptophan reduced the severity of DSS-induced colitis in pigs [57].

Again, intestinal microbiota play a fundamental role in the beneficial outcomes of dietary tryptophan. In the body, tryptophan is subject to biosynthetic manipulation by the host and microbes to generate various metabolites [58]. Specifically, ingested tryptophan is metabolised in the intestines by three distinct pathways; the first two of these pathways—the serotonin and kynurenine pathways—are conducted by host enzymes, whilst the third pathway, which produces the immunomodulatory indole compounds, is performed by microbiota [59].

A broad-spectrum of indole compounds is generated by the intestinal flora—the structure of each metabolite differing according to the biochemical transformations they are subjected to. *Clostridium sporogenes*, from the phylum *Firmicutes* for example, generates indole-propionic acid from tryptophan through induction of the enzyme phenyllactate dehydratase [60]. Moreover, *Lactobacilli reuteri* generates indole-3-aldehyde from tryptophan—a reaction catalysed by an aromatic amino acid aminotransferase [61]. Alternatively, indole may also be sourced directly from the diet from produce such as broccoli, which similarly has been shown to attenuate murine colitis [62].

Encouragingly, indole compounds have also been reported to promote mucosal homeostasis in the intestines [63]. The mechanistic basis for indole alleviating intestinal inflammation can be partly attributed to interactions with the Aryl hydrocarbon receptor (AHR), which various indole compounds display affinity for [64]. The AHR is a transcription factor that upon activation is responsible for several anti-inflammatory mechanisms, including regulating intestinal homeostasis. In macrophages, AHR dampens LPS-induced inflammation—as mice lacking macrophage-specific expression of AHR are more susceptible to LPS-induced septic shock [65]. Given that macrophages propel inflammatory signalling in IBD, whether interactions between indole and macrophages can modulate cellular plasticity is an avenue of interest.

Interestingly, indole compounds have previously been shown to suppress macrophage inflammatory signalling in the context of treating liver disease. Notably, Krishnan et al. reported that pre-treating macrophages with microbial-derived tryptophan catabolite indole-3 acetate (I3A) attenuates production of TNF-α, IL-1β, and monocyte-chemoattractant protein-1 (MCP-1) in response to LPS and palmitic acid [66]. Similarly, Zhao et al. detailed that pre-treating murine J774A. 1 macrophages with indole-3-propionic acid lessened secretion of IL-1β, TNF-α, and IL-6 via repression of NF-ĸB [67]. Taken together, these results suggest that indole compounds act to promote a more tolerogenic macrophage phenotype by decreasing responsiveness to inflammatory stimuli. Therefore, it is not beyond the realm of possibility that similar outcomes may arise to attenuate inflammatory signalling in the intestines.

Several bodies of evidence give the impression that indole–AHR interactions may be crucial for maintaining intestinal mucosal immunity. Firstly, AHR activation negatively regulates production of pro-inflammatory TNF-α and IL-6 [68]. Next, expression of AHR has been documented to be diminished in patients with IBD [69]. Furthermore, indole compounds have been shown to act as agonists of AHR [64] Collectively, these studies illustrate that a microbiota-targeted approach aiming to optimise binding of indole compounds to AHR may be an attractive target for future IBD therapies.

More importantly, this work has evinced the potential of tryptophan metabolites as another addition to the armoury for modulating disease course in IBD. Considering the diverse signalling landscape in IBD, these metabolites could be highly beneficial by virtue of an ability to target immune cell populations and induce downstream modulation of intestinal epithelial cell function. Although it is apparent that indole compounds mediate immune altering effects, the question remains whether these findings can be translated to a meaningful therapy for IBD. For this to occur, we firstly need to identify means to prolong bioavailability of indole compounds in the body, such as by increasing production, or by reducing consumption by the microbiota. Accordingly, future studies should validate the predominant tryptophan-metabolising microbiota, along with identifying the most potent indole compounds. Importantly, further studies should be attentive to whether indole compounds can alter tissue-resident macrophage plasticity, which has not been studied. 

### 3.3. Omega-3 Polyunsaturated Fatty Acids

Dietary fats are generally viewed as negative elements of the Western diet—central to the pathogenesis of a wide range of diseases. However, in recent years, studies are beginning to portray fatty acids in a more favourable light, as the beneficial impact of certain fatty acids on intestinal health become apparent. The ω3FAs, such as eicosapentaenoic (EPA), docosahexaenoic acid (DHA), and α-linolenic acid, are abundant in oily fish, nuts, seeds, and plant oils.

Adherence to the Mediterranean diet, which is rich in foods with high levels of ω3FAs (seafood, seeds, nuts, and olive oil), has been closely interlinked by epidemiological studies to lessened risk of many inflammatory diseases, including IBD [70]. Notably, meta-analysis of twelve studies that measured fish consumption revealed an inverse relationship between dietary intake of fish and risk of IBD [71]. In a similar manner, dietary supplementation of extra-virgin olive oil and canola oil, both containing α-linolenic acid, alleviated gastrointestinal symptoms and reduced inflammatory markers in patients with UC [72].

When ingested, ω3FAs serve as precursors for synthesis of anti-inflammatory mediators such as resolvins, protectins, lipoxins, and moresins, which dampen inflammation and protect against organ damage [73]. Accordingly, clinical studies have studied these ω3FAs with the aim of elucidating involvement in intestinal disease. In a study of patients with UC, Pearl et al. showed lower levels of ω3FAs were present in colonic mucosa biopsy samples, suggesting dysfunctional fatty acid metabolism may be involved in exacerbating disease [74]. Considering the enormous therapeutic potential of harnessing the immunomodulatory properties of ω3FAs, various laboratories have attempted to characterise the precise mechanisms underpinning their anti-inflammatory signature.

Interactions with the microbiota is one such means that ω3FAs engage in anti-inflammatory activities and facilitate intestinal homeostasis. Studies have uncovered that ω3FAs mediate positive effects by altering microbiota diversity. Indeed, this effect encompasses both increases in beneficial bacteria, along with reductions in populations of harmful bacteria. Notably, ω3FA encourages growth of SCFA-producing microbes, including the *Lachnospiraceae* family of bacteria [75]. On the other hand, ω3FA supplementation in infants led to lessened abundance of pathogenic microbes, such as *Enterobacteriaceae* [76].

The anti-inflammatory profile of dietary ω3FA may also be altered by biosynthetic manipulation by the microbiota. For example, α-linolenic acid is metabolised by *Lactobacillus plantarum AKU 1009a* to generate the bioactive metabolite 10-oxo-cis-12-cis-15-octadecadienoic acid (α-Keto acid) [77]. Interestingly, pioneering research has shown that anti-inflammatory properties are retained by α-Keto acid [78]. The authors revealed that α-Keto acid was capable of inducing M2 macrophage polarisation in mice administered a high-fat diet.

Omega-3 fatty acids are also postulated to modulate macrophage function through interactions with the peroxisome proliferator-activated receptors (PPARs), including PPAR-α, PPAR-β/δ, and PPAR-γ. PPARs are a family of transcription factors involved in cellular differentiation and metabolism [79]. PPARα interacts with both EPA and DHA, leading to modulation of genes central to glucose and lipid homeostasis [80]. Activation of PPAR-α promotes FAO, one of the preferred metabolic pathways for shaping M2 macrophage responses [80]. The relationship between ω3FAs and PPAR-γ, which is master of lipid homeostasis, is also of heightened interest, as ligation of PPAR-γ skews macrophages toward an M2 phenotype and inhibits NF-ĸB [81]. Consequently, pro-inflammatory mediators are reduced, characterised by suppression of prostaglandin E2 (PGE2), TNF-α, IL-6, and IL-1β release [82,83].

Several studies have focused on the modifying effects ω3FAs exert on macrophages, which sense and respond to other dietary components. It appears that these responses extend to ω3FAs, as macrophages cultured in the presence of EPA exhibited lessened production of TNFα and IL-6 induced by LPS challenge, whilst displaying upregulated secretion of IL-10 [84]. Moreover, in a study involving krill oil, which contains high levels of EPA and DHA, application of krill oil attenuated LPS-induced secretion of TNF-α, IL-6, and IL-1β through regulation of NF-ĸB and nucleotide-binding oligomerization domain (NOD)-like receptor signalling [85]. While LPS downregulated PPAR-γ, a marker of M2 polarisation, this was reversed by krill oil treatment, which is favourable for directing macrophage plasticity toward an M2 phenotype. However, despite ω3FA inducing macrophage responses consistent with anti-inflammatory reprogramming, neither study carried out macrophage phenotyping or metabolomics following ω3FA treatment, which could be the basis of further studies.

Other studies conducted investigations using in vivo models of inflammation, with rodent and porcine models generally being the preferred approaches. In a DSS-induced model of colitis in rats, the ω3FA α-linoleic acid, found in various seeds and oils, was capable of abrogating disease markers of intestinal inflammation, as illustrated by downregulated expression of IL-1β and TNF-α [86]. Similar conclusions were drawn by Arisue et al., who showed that fish oil ameliorates intestinal injury and proposed that this may occur through downregulation of oxidative stress [87]. Furthermore, in a porcine model, where animals were challenged with LPS, piglets administered a diet supplemented with fish oil showed reduced intestinal NF-ĸB protein and TLR4 expression, along with diminished TNF-α secretion [88]. Future research could broaden the scope of this study by mapping gene expression back to individual intestinal cell populations using novel transcriptomic technologies. Single-cell RNA sequencing could permit us to determine inflammatory signatures of the intestinal cell repertoire and delineate the effects of ω3FA on macrophage plasticity.

Collectively, data obtained in these studies highlight that ω3FAs are an encouraging prospect as an adjuvant for IBD therapy. Nonetheless, interplay between tissue-resident macrophages and ω3FAs remains relatively unstudied. Questions remain regarding whether these metabolites can modulate responses and transcriptional activity of intestinal macrophages populations, which warrants further research. Moreover, considering the importance of metabolic programming in governing cellular responses, interactions between ω3FA and PPAR-γ, which are central in lipid metabolism, are worthy of further research.

## 4. Future Therapeutic Perspectives

### 4.1. Novel Therapeutic Approaches

While it is well established that macrophages are crucial drivers of tissue destruction and fibrinogenesis in IBD, therapies specifically targeted at macrophages are limited. Ideally, future therapies would be directed to dampen macrophage inflammatory signalling, whilst halting tissue fibrosis that progressively impairs organ function.

While several studies have documented several microbiota-derived metabolites possess immunomodulatory properties, little is known regarding the extent to which these alter macrophage plasticity and metabolism. Targeting macrophage plasticity is a novel approach that has gained traction in recent years, and there is a strong rationale for determining whether dietary inventions can control these molecular switches. Encouragingly, therapies involving dietary supplementation of SCFAs, indole-compounds, or ω3FAs could provide a safe approach for attenuating intestinal injury.

However, questions remain regarding the clinical efficacy and gut bioavailability of these molecules. To address these potential limitations, we should consider means of optimising cellular-specific delivery and limiting degradation along the gastrointestinal tract. Firstly, development of a framework for identifying patient subtypes that respond best to each intervention, along with clarifying targeted therapies that metabolites are most effective in combination with, would be highly beneficial. Together these could serve as a basis for minimising non-responders to therapy and optimising clinical efficacy. While these metabolites may not represent an approach for completely halting inflammatory signalling and intestinal injury, they offer a means of raising the therapeutic ceiling of more established interventions—a notable example being SCFA supplementation enhancing targeted therapy against TNF [89]. Moreover, we could benefit greatly from studying molecular interactions of these molecules with the epigenome and intracellular signalling pathways, as this could enlighten us toward novel targets for pharmacological interventions.

Another potential benefit of dietary-based approaches for IBD is that there may be scope for achieving synergistic effects when administering interventions concurrently. Synergistic effects can be defined as when multiple treatments interact to reach an overall effect greater than each treatment on an individual level. While previous studies have primarily focused on individual metabolites, combined dietary-based approaches could offer a means of targeting multiple anti-inflammatory pathways simultaneously, which could accentuate treatment efficacy. SCFAs for example, heighten responsiveness of the AHR in colonocytes [90], indicating that co-application of SCFA and indole compounds could represent a promising approach for IBD therapy. Further studies are warranted to establish synergism that may arise from therapies targeting the microbiota. 

### 4.2. Novel Technologies

Specific delivery of these molecules toward macrophages would be crucial for optimising therapeutic efficacy. Recent advances in nanomedicine have led to development of novel drug delivery systems. Nanoparticles, for example, may provide an approach for macrophage-specific drug delivery for treatment of inflammatory disease [91]. Therefore, encapsulation of metabolites within nanoparticles could be a strategy for tailoring macrophage-specific therapies. Moreover, modifying the microbiome to optimise metabolite bioavailability is another approach that should be considered. In this context, treatments could involve supplemented tryptophan/SCFA/ω3FA metabolites with precise microbiome modulating interventions. 

Ultimately, previous findings support the concept of applying dietary-based therapies for promoting the activation of anti-inflammatory mechanisms in the gut. Interest in this area has been rapidly growing as we comprehend how far macrophage involvement extends within the pathogenesis of inflammatory disease. Although this is a relatively new area of study, the field is gaining great momentum by virtue of the discovery of novel approaches for studying immune cell interactions in greater detail. Several developments in the area of mass cytometry and transcriptomics have revolutionised our understanding of complex cellular heterogenicity. Spatial transcriptomic technologies, for example, have been employed to uncover tissue-wide heterogenicity in cancer research. Evidently, there is a large scope for discovery through application of this technology to characterise the inflammatory signature of macrophages in diseased tissue. Future studies should prioritise applying these technologies to IBD research, ensuring that such breakthroughs could reveal a clearer picture of how macrophages propel the dysregulated tissue architecture prevalent in IBD [92].

## 5. Conclusions

Collectively, there is compelling evidence for employing microbiota-derived metabolites to target macrophage plasticity for IBD therapy. While the entire repertoire of molecular signalling pathways that co-ordinate macrophage responses to microbiota-derived metabolites has not yet been determined, we can be assured that our knowledge of this fascinating interplay is rapidly growing. All things considered, macrophage-specific targeted therapies are a novel approach that could revolutionise the treatment of inflammatory disease in the future.

## Data Availability

Not applicable.
